# Editorial: Combinational immunotherapy of cancer: novel targets, mechanisms, and strategies

**DOI:** 10.3389/fimmu.2023.1250975

**Published:** 2023-08-11

**Authors:** Yanyang Nan, Dianwen Ju, Xuyao Zhang

**Affiliations:** Department of Biological Medicines & Shanghai Engineering Research Center of Immunotherapeutics, School of Pharmacy, Fudan University, Shanghai, China

**Keywords:** cancer immunotherapy, combination therapy, novel targets, therapy strategies, immune checkpoint inhibitors

Cancer immunotherapy, distinct from traditional cancer therapies, is achieving unprecedented success by harnessing the host’s immune system to control tumor progression. Current clinical strategies for cancer immunotherapy include immune checkpoint inhibitor therapy, chimeric antigen receptor T-cell therapy, oncolytic virotherapy and tumor vaccines (1–4). Nevertheless, the effectiveness of immunotherapy varies significantly among patients, with only a minority experiencing long-term clinical benefits (5). This highlights the need to identify potential targets of tumor therapy and diversify our combinational immunotherapy strategies, as well as to unravel the underlying molecular events.

Because the critical for tumor markers, researchers are dedicating to uncover novel biomarkers that can be used to identify cancer in its early stages, and to predict the effectiveness of treatment and the chance of cancer recurrence. Chen et al. disclosed the potential role of Glypican 2 (GPC2) in multiple cancers *via* pan-cancer bioinformatical analysis. Their data identified that GPC2 expression in multiple cancer types was significantly higher than that in normal tissues. High diagnosis performance of GPC2 was discovered in 6 types of cancer, and immune-related genes were highly co-expressed with GPC2 in 33 tumors, illuminating GPC2 can be used as a promising diagnostic, prognostic, and immunological biomarker in tumor. Wu et al. demonstrated that Apolipoprotein E (ApoE), which was secreted from melanoma cells, has an immune suppressant effect by inducing the secretion of IL-10 from activated dendritic cells and further suppressing T-cell function partially *via* the lrp8 receptor pathway. Moreover, ApoE knockout induced significant tumor suppression and improved overall survival in mouse melanoma model, hence providing a potent strategy for cancer immunotherapy by targeting ApoE. Squalene epoxidase (SQLE) is a key enzyme in regulating cholesterol metabolism. You et al. disclosed the upregulated expression of SQLE in pancreatic adenocarcinoma (PAAD) patients with poor disease-free survival and overall survival. Comprehensive analyses of multiple bioinformatic databases and anti-PD-1 clinical trials showed that the expression of SQLE was strongly negatively correlated with checkpoint inhibitors, immune infiltrations, and immunotherapy outcome. This study provides a promising target to potentiate the efficiency of immunotherapy in PAAD.

Although inhibitors targeting immune checkpoints have demonstrated efficacy in specific patient subgroups, optimal use is encumbered by high rates of drug resistance. To further enhance the antitumor effects of the existing cancer immunotherapies, researchers have performed various explorations. Wang et al. took an in-depth look on the drug resistance mechanisms of colorectal cancer (CRC) during anti-PD-1 treatment. Innovatively, the authors revealed that proprotein convertase subtilisin/kexin type 9 (PCSK9), a lipid metabolism-related protein, was upregulated after anti-PD-1 treatment. Targeting PCSK9 with anti-PCSK9 antibody enhanced antitumor effect of anti-PD-1 monotherapy by increasing both the infiltration of CD8^+^T cells and release of inflammatory cytokines, as well as reducing the proportion of Treg cells in tumor microenvironment. This study proposed a novel combinational immunotherapy strategy to overcome anti-PD-1 resistance in CRC by simultaneously targeting PD-1 and PCSK9. Vitale et al. engineered a novel oncolytic adenovirus expressing an anti-PD-L1-scFv based on Ad5Δ24 adenovirus (Ad5Δ24-anti-PD-L1-scFv) to improve the antitumor activity of oncolytic virotherapy in melanoma. Ad5Δ24-anti-PD-L1-scFv not only secreted anti-PD-L1-scFv blocking PD-1/PD-L1 pathway, but also induced cytopathic and lytic effects in melanoma cells. Moreover, intra-tumor injection of Ad5Δ24-anti-PD-L1-scFv enhanced the infiltration of CD8^+^T cells and effectively inhibited tumor growth. Although tumor vaccines based on dendritic cells (DCs) play a key role in tumor immunotherapy, the poor immunogenicity and weak immune response rate still limit their efficacy (6). Zeng et al. constructed a novel DCs-based therapeutic vaccine titled MSLN-PDL1-GMCSF that could self-activate and induce anti-PD-L1 antibody while targeting MSLN. The MSLN-PDL1-GMCSF vaccine elicited a robust and specific immune response in lung cancer. Moreover, combining the vaccine with PD-1 blockade demonstrated a synergistic antitumor effect, presenting a promising and effective combination strategy for tumor immunotherapy. Zhou et al. summarized and reviewed the underlying resistance mechanisms of immune checkpoint blockade (ICB), particularly in relation to the biological function of CD8^+^ T cells, and compiled a comprehensive overview of the latest combination strategies to enhance the effectiveness of ICB treatments.

In summary, the articles included in the project “*Combinational Immunotherapy of Cancer: Novel Targets, Mechanisms, and Strategies*” not only describe potential targets to expand our toolbox for manipulating antitumor immunity, but also provide novel combinational strategies to enhance antitumor therapy responses. Persisting investigation of new targets and combinational strategies could result in a better understanding of antitumor treatments and provide valuable promises for tumor immunotherapy.

## Author contributions

YN, and XZ drafted the manuscript. XZ and DJ contributed to the design and critical revision of the manuscript. All authors contributed to the article and approved the submitted version.
